# A Novel Strategy to Enhance Antioxidant Content in *Saccharomyces Cerevisiae* Based on Oxygen Pressure

**DOI:** 10.3390/bioengineering10020246

**Published:** 2023-02-13

**Authors:** Na Cui, Patrick Perré, Emilie Michiels, Victor Pozzobon

**Affiliations:** Université Paris-Saclay, CentraleSupélec, Laboratoire de Génie des Procédés et Matériaux, Centre Européen de Biotechnologie et de Bioéconomie (CEBB), 3 rue des Rouges Terres, 51110 Pomacle, France

**Keywords:** flow cytometry, reactive oxygen species, antioxidant properties, phenolics, ferrous ion chelating

## Abstract

Antioxidant foods represent a potent lever to improve diets while creating value. Yet, their cultivation is often tied to a specific area and climate, limiting availability and increasing market cost. Therefore, microorganism-based antioxidant production emerges as a promising technology to solve these problems. In this view, a novel process was investigated for antioxidant accumulation in yeast culture. *S. cerevisiae* cells were exposed to various hyperbaric air conditions from 1 to 9 bar (A). Yeast cultures exhibited an increased reactive oxygen species content, which induced oxidative defense expression. After a few hours, reactive oxygen species levels decreased while antioxidant contents remained high, leading to a net increase in antioxidant power. At 6 bar (A), yeast achieved the highest net antioxidant power (phenolics content +48.3 ± 18.6 %, reducing power +120 ± 11.4 %) with an acceptable growth rate (0.27 h^−1^). Regarding time evolution, a 2 h exposure seems to be the optimum: cells have the lowest reactive oxygen species level while their antioxidant power is increased. From a biotechnological perspective, this finding highlights air pressure as an antioxidant-manipulating stress strategy. Moreover, the proposed process led to a patent that could potentially reduce energy and chemical consumption in such antioxidant accumulation processes.

## 1. Introduction

Functional foods and nutraceuticals have the potential to improve human health status as well as support economic growth in rural communities. A significant focus of the functional foods and nutraceuticals market is antioxidants, as damages caused by reactive oxygen species (ROS) are the basis for aging and several diseases, including chronic kidney disease, cardiovascular disease, and cancer [1,2,3]. It is well documented that several traditional herb and plant extracts have antioxidant properties, which are potential candidates to prevent and treat ROS-induced diseases [4,5,6]. One of those herbs is goji berry, whose extracts have been shown to protect from damage caused by ROS [7], such as oxidative stress-induced disorders (such as neurological) and pathological conditions (such as aging) [8].

Conventional production of antioxidants is generally performed by maceration, such as from apple pomace [9], or extraction by organic solvents, such as methanol or acetone extraction from red fruits [10]. This technique is not always interesting for industrial production because the process is slow, expensive, requires toxic extraction, and demands costly technological aids, such as purification or removal of toxic solvents. The sustainability of these processes is also questionable in terms of the principles of green chemistry [11].

An alternative way to produce antioxidants is to use microorganisms (fungi, yeast, microalgae, etc.). This approach features several benefits. Unlike synthetic antioxidants, microorganisms-based ones are nontoxic and noncarcinogenic [12]. Compared to natural antioxidants, their production is not tied to a specific area and climate, limiting availability and increasing market cost, as fermentation can be led anywhere in the world [13]. Costs can be further reduced by growing microorganisms on alternative substrates, such as food-grade potato waste [14,15]. In addition, their use is intended as direct inclusion into food and feed [12,16,17], limiting the need for post-harvest treatment and extraction. In this view, the integration of yeast or microalgae in animal diets has been proven to improve major zootechnic traits (immune system, gastrointestinal tract functioning, lean mass gain, etc.) [18,19,20]. Focusing on antioxidants, yeast and yeast extract have been acknowledged as a sources of bioactive molecules. Adding these compounds can prevent the oxidation of food products, such as fats and baby foods, after a series of organic extractions of yeast suspension [21]. Indeed, owing to their antioxidant properties, yeast is regarded as a feed supplement of choice for aquaculture [22], and protein and pharmaceuticals for humans [23].

Classically, when an increase in cell antioxidant content is sought after, microorganisms are cultivated under stress. Several methods can be employed to cause stress to microorganisms. It is known that adding hydrogen peroxide (H_2_O_2_) to the culture generates stress on the microorganism [24,25]. However, this method needs the use of H_2_O_2_ in the growth process to be approved by competent authorities (e.g., US FDA, EU EFSA), primarily when the microorganisms are intended for food or feed. From a more general perspective, in the case of implementing the toxic chemical molecule, the latter must necessarily be removed from the medium before using the biomass, and such a step of separating a chemical species is costly and time-consuming.

Another method known in the prior art for increasing the antioxidant power of microorganisms is to transfer the culture to a medium deficient in an element essential for growth. In the nitrogen stress approach, such a transfer can thus be made, for example, from an initial culture medium containing nitrogen in sufficient quantity for the multiplication of the microorganisms to a second medium that is nitrogen-deficient [26]. However, before the transfer from the initial culture medium to the deficient one, it is necessary to perform a culture wash step. Again, there are costs involved in performing this step, as the microorganisms must be separated from the initial culture medium. Another solution is to manipulate the initial quantities of the nutrients. In this case, the quantity of nitrogen has to be calculated extremely precisely when preparing the initial culture medium. The objective here is to avoid a transfer step as the culture medium becomes naturally deficient. However, such a solution remains complicated to implement, particularly at the industrial scale.

Hence, in this work, a particular focus was set on remedying, at least in part, the disadvantages of the above-mentioned processes by proposing a solution implementable in culture or maturation bioreactors without adding supplementary chemical products.

This method is based on the dual role of O_2_ in cell metabolism. Paradoxical to the oxygen requirement for cellular respiration, oxygen can cause cell degeneration by attacking cellular compounds, such as DNA, lipids, sugar, and proteins [1]. In dire cases, O_2_ can even lead to programmed cell death by the generation of ROS, such as superoxide (•O_2_^−^), hydrogen peroxide (H_2_O_2_), and hydroxyl radical (•OH). Yet, under normal physiological conditions, cells can maintain a stable intracellular redox environment. To do so, cells express antioxidants to regulate energy metabolism and material metabolism and keep cellular homeostasis. This study consists of exposing microorganisms to a high oxygen pressure (contained in the air) during their cultivation to trigger well-calibrated oxidative stress leading to antioxidant upregulation while avoiding cell death. 

As a model microorganism, this research opted for yeast, which is already authorized and used in the feed and food industry. Singularly, to date, no authors have studied the potential techniques of increasing antioxidants in yeast cells by employing oxygen stress. To lead this work, *S. cerevisiae* was cultivated using a modified YPD medium with oxygen applied through air pressure under 1, 4, 6, 8, and 9 bar (A). The monitored outcomes were growth rate, ROS, and antioxidant assays. Conveniently, the pressurization process is easy to deploy, and no chemicals need to be added, thus avoiding purification costs. This convenient invention has been patented [27].

## 2. Materials and Methods

### 2.1. Strain and Subculturing

The strain used for this study was budding yeast *S. cerevisiae* FIZZ+ from a dry commercial yeast facility Œnologique de Champagne (IOC, Epernay, France). Cells were subcultured using YPD agar stock medium at 25 °C for 5 days and subsequently stored at 4 °C before subculture. This solid YPD medium was composed of 20 g/L D-glucose, 10 g/L yeast extract, 20 g/L peptone, and 15 g/L agar. The stock plates were replaced every 3 months. 

Yeast cells were amplified in 500 mL Erlenmeyer flasks filled with 150 mL of liquid YPD medium (20 g/L D-glucose, 10 g/L yeast extract, 20 g/L peptone) at 125 rpm of shaking rate and a temperature of 25 °C.

### 2.2. Batch Cultivation and Experimental Setup

**Figure 1:**
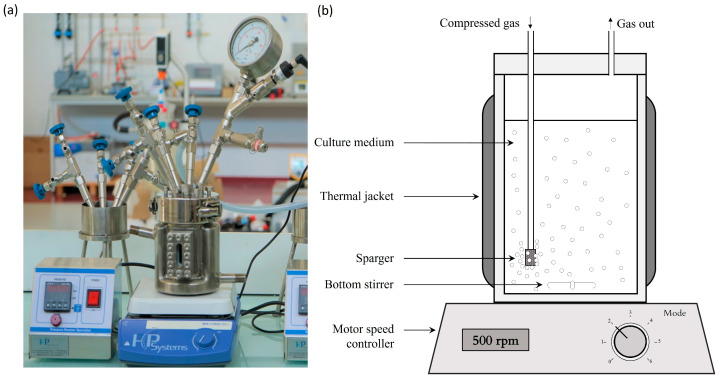
Photo (**a**) and diagram (**b**) show the aerated bioreactor configuration used for the culture of *S. cerevisiae*. Compressed air is injected through the sparger to pressurize the medium in the bioreactor and to supply oxygen. Gas leakage from the outlet was included to maintain the gas flow to provide a sufficient supply of oxygen. The temperature was maintained by a double jacket, and the agitation was achieved by a magnetic stirrer.

**Figure 2:**
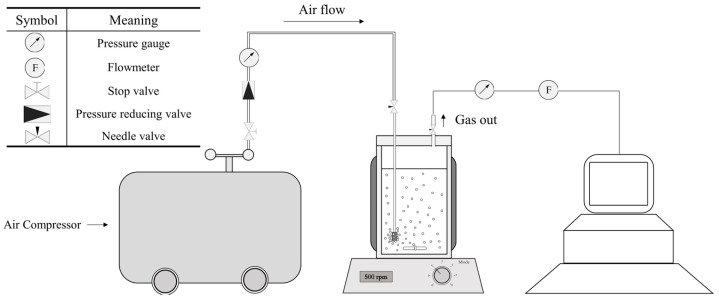
Air pressure apparatus and symbols. Compressed air is supplied by an air compressor. The pressure and airflow were controlled by a gas flow meter and pressure reducer, respectively. Online pressure was monitored by a pressure sensor.

Special pressure-resistant (up to 9 bar (A)) bioreactors were used for this study (Figure 1a,b). They can host a working volume of 500 mL (750 mL total volume). A magnetic stirrer (500 rpm) was used to ensure proper agitation of the culture medium. Air was constantly blown through a sparger into the bioreactor (0.8 NL/min, 1.6 VVM). The temperature was regulated at 25 °C thanks to a water circulation into a double jacket surrounding the culture volume. The pressure was set by a system designed to measure and control the pressure inside of the bioreactor (Figure 2). Compressed air (21% O_2_) was used to pressurize the system. The operating pressure was adjusted by manipulation of the pressure of the inlet gas and the adjustable valve position of the exit gas line. In addition, a pressure transducer was fitted to the bioreactor. It was used to monitor the total internal pressure over time and validate that the entire experiment was achieved under the specified conditions. Finally, adequate oxygen supply to the cell was demonstrated by oxygen transfer and oxygen uptake rates computation in our preliminary work [28].

To lead a culture, the bioreactors were filled with 500 mL of modified YPD (8 g/L D-glucose). An initial cell concentration of 0.3 g/L was used for inoculation with 24-h-old inoculum. Cultures were stopped after 8 h, corresponding to glucose exhaustion. The study was carried out in biological triplicates in axenic conditions.

### 2.3. Cell Growth Measurement

Samples were drawn out of the bioreactors about every two hours. Before each sample, a fraction of the culture (around 5 mL) was sampled and discarded in order to purge the sampling duct. Then six samples were taken from each bioreactor. One 5 mL sample was used for the analysis of the cell population (dry weight in g_DW_/L and microscope observation), and five 1.9 mL samples were frozen immediately in liquid nitrogen to lead antioxidant assays. Over a run, about 20% of the culture was withdrawn from a bioreactor (including the effluent discharged from the sampling tube), which ensured that the growth conditions were kept almost constant from a hydrodynamic perspective.

### 2.4. ROS Assay

The ROS assays were conducted with a Sysmex CyFlow Space flow cytometer according to the method introduced by Pozzobon et al. [29]. The ROS content in the cells was measured using 2,7-dichlorodihydrofluorescein diacetate (H_2_DCFDA). This dye can penetrate the cells and emit fluorescence at a level proportional to the intracellular amount of ROS.

This flow cytometer was mounted with a blue laser (488 nm), forward scatter (FSC) and side scatter (SSC) detectors, as well as three fluorescence channels (FL1 to FL3, 536/40, 590/50, and 675/30 nm, respectively). This setup allowed us to acquire three different signals from unstained yeast cells: size (FSC) and complexity (SSC). FDA identifies the intensity of the ROS in the living cells on channel FL1. The values were normalized by the value at t = 0 h.

### 2.5. Sample Preparation for Antioxidant Assays

The frozen yeast pellet is resuspended in 1 mL of cold double-distilled water (ddH_2_O) and 250 μL of glass microbeads. Yeast cells were broken by a high-speed benchtop homogenizer (MP Biomedicals™ FastPrep-24™ 5G Instrument, Fisherbrand, Waltham, MA, USA) at 6.5 m/s with 5 cycles and a 60 s pause in-between. Then, the samples were centrifuged at 10,000 rpm for 10 min, and the supernatants were used to perform the antioxidant assays. The reported protocols are a variation of the methods compiled by Kesraoui et al. [30] (namely: total phenolics [31], reducing power [32], ferrous ion chelating activity [33], and β-carotene-linoleic acid assay [34]). During the current protocols, only the key variations and the concise process are presented in Section 2.6, Section 2.7, Section 2.8 and Section 2.9. ddH_2_O was used as the negative control of all the assays. In order to be certain that the variation in antioxidant assays was due to real biological changes, the results were normalized by the value at t = 0 h.

### 2.6. Determination of Total Phenolics

A 50 µL sample was mixed with 125 µL of 0.2 M Folin–Ciocalteau reagent and 700 µL of ddH_2_O. After 5 min at room temperature, 125 µL of 16% (*w*/*v*) Na_2_CO_3_ was added. Sample absorbance was read at 760 nm (UV-visible spectrophotometer Cary 100 Conc, Varian, Palo Alto, CA, USA) after 1 h reaction time at room temperature. For this test, gallic acid was used as the standard.

### 2.7. Reducing Power Assay

A 50 µL sample was mixed with 50 µL of phosphate buffer (0.2 M, pH 6.6) and 50 µL of 1% (*w*/*v*) K_3_Fe(CN)_6_. The solution was left to react for 20 min at 50 °C. Test solutions were then cooled, and the reaction was neutralized by adding 50 µL of 10% (w/v) trichloroacetic acid (TCA), vortexing, and pelleting the sample. A total of 200 µL of the upper layer mixture was recovered and mixed with 200 µL of ddH_2_O and 40 µL of 0.1% (*w*/*v*) FeCl_3_. After 10 min at room temperature, the absorbance of the mixture was read at 700 nm. For this test, vitamin C was used as a positive control.

### 2.8. Ferrous Ion Chelating Activity Assay

A 300 µL sample was mixed with 50 µL of 2 mM ferrous chloride tetrahydrate (FeCl_2_•4H_2_O) and 300 µL of ddH_2_O. A total of 100 µL of 5 mM ferrozine was then used to start the reaction. The absorbance was read at 562 nm after 10 min of reaction at room temperature. Ethylenediaminetetraacetic acid (EDTA) was used as a positive control. The sample chelating effect (for Fe^2+^ ions) ion was calculated based on Equation (1):

Chelating effect (%) = ((A_Ctrl_ − A_Sample_)/A_Ctrl_) × 100 (1)

where A_Ctrl_ and A_Sample_ are the absorbances of the positive control and the sample, respectively.

### 2.9. Antioxidant Activity by β-Carotene-Linoleic Acid Assay

To prepare the β-carotene reference solution, 2 mg of β-carotene was first dissolved into 10 mL of chloroform. Then, 40 mg of linoleic acid and 400 mg of tween 40 were added to 4 mL of the β-carotene solution. Chloroform was evaporated at 40 °C under a vacuum before adding 100 mL of oxygenated water. The mixture was vortexed extensively. To carry out the tests, the same amounts of sample and phenolic extract were mixed (500 µL in total). The mixture was then incubated at 50 °C for 2 h (agitated water bath). Two different measurements were led per sample: one a t = 0 h, to serve as a reference; one after 2 h of reaction. For both, the absorbance was read at 470 nm. BHT was used as a positive control for this test. The antioxidant activity was calculated according to Equation (2):

Antioxidant activity = (A_2h_/A_0_) × 100 (2)

where A_2h_ is the absorbance of β-carotene after 2 h of incubation and A_0_ is the absorbance of β-carotene at the beginning of the reaction. 

## 3. Results and Discussion

### 3.1. Effect of Different O₂ Pressures on the Growth Rate of S. Cerevisiae

**Figure 3:**
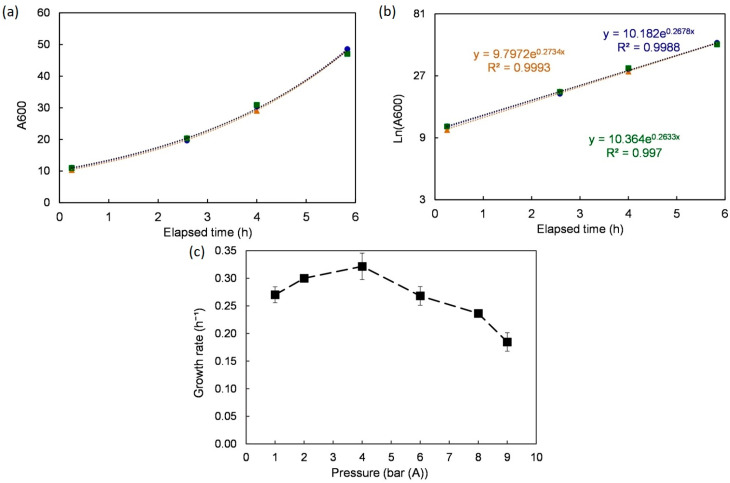
Growth rate under different pressures. (**a**) One example (atmospheric pressure) shows the time evolution and the quality of repetition. (**b**) Log scale of the same example to extract the growth rate. (**c**) Effect of pressure on the growth rate. Data are shown as average ± standard deviation (n = 3).

First, the growth rates under different pressures were analyzed (Figure 3). Under atmospheric pressure, *S. cerevisiae* grew exponentially (µ = 0.27 ± 0.01 h⁻¹) over the first 8 h following inoculation. A semi-log plot was used to both confirm the observation (R^2^ > 0.99) and extract the growth rate value using linear regression. The same method was applied to the growth curves obtained under different tested pressures (2, 4, 6, 8, and 9 bar (A)). Once again, growth rates were computed using linear regression over the relevant time ranges. The yeast grew well up to 6 bar (A) with performances superior or equal to the ones under 1 bar (peak at 4 bar, µ = 0.32 ± 0.02 h^−^¹). For higher pressures (8 and 9 bar), the growth rate started dropping with a moderate trend from 6 to 8 bar and sharply afterward (at 9 bar, µ = 0.18 ± 0.21 h⁻¹). These results are consistent with observations reported previously [28].

### 3.2. ROS Content

**Figure 4:**
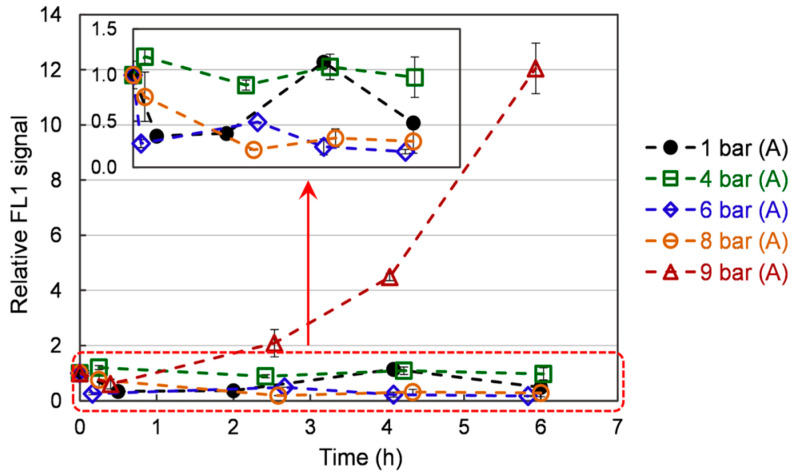
The ROS evolution in *S. cerevisiae* cells over time under different pressures. Data are shown as average ± standard deviation (n = 3).

Figure 4 represents the ROS content in the cells over time, depending on the pressure applied. ROS intensity increases slightly with respect to time zero immediately after the start of the application of the hyperbaric conditions on the culture. Over the culture, the ROS content exhibits a general declining trend (the point at 4 h for the 1 bar case is deemed an artifact). This trend is well marked under 1 bar (A), 6 bar (A), and 8 bar (A), with values decreasing from 1 to 0.5 ± 0.1, 0.4 ± 0.1, and 0.4 ± 0.1, respectively. The 4 bar (A) has a constant ROS intensity over time (with respect to the measurement error). On the contrary, it increases from 1 to 12 ± 0.90 under 9 bar (A). The observed trends can be related to what is known about ROS generation in animal models. It has been shown that mitochondria naturally generate ROS, even under normal conditions [35]. Furthermore, acute hyperbaric oxygen pressure (e.g., repetitive exposure 2 bar of pure oxygen for 2 h) has been reported to increase tissue ROS content and induce oxidative damage [36]. This increase can be linked to ROS overproduction by the mitochondria. While this condition leads to premature aging (myopia, cataracts) in animal models, it also upregulates the antioxidant content of the exposed tissues [37]. Still, the authors also report that this increase in antioxidants fails to lower ROS content [37]. In our case, the treatment is much more moderate. It can be hypothesized that yeasts overexpress antioxidants (see following sections) and successfully lower their ROS content. Still, when the pressure is too high (9 bar), the amount of generated antioxidants is not important enough to quench ROS. Consequently, an ever-increasing ROS content is observed. Furthermore, cellular damages may also be induced, leading to the substantially lower growth rate observed under 9 bar.

### 3.3. Total Phenolics

**Figure 5:**
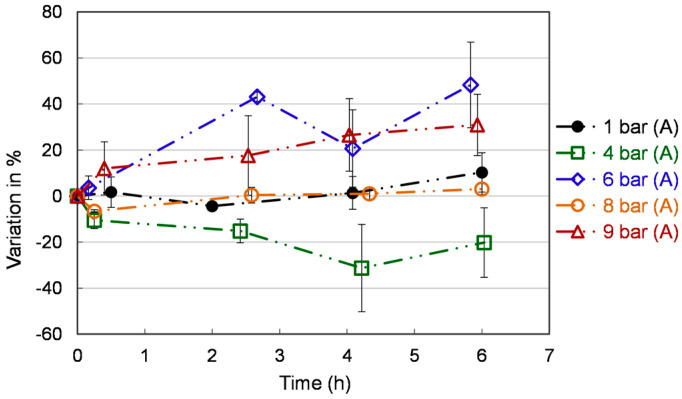
Total phenolics content in *S. cerevisiae* extracts under different pressures. Each value is expressed as the average ± standard deviation (n = 3).

Figure 5 reports the variation of the total phenolic yield with respect to the time zero. At the beginning of the experiments, the total phenolic contents were 113 ± 50 mgGAE/100 g, which aligns well with Vieira et al. findings (113 mgGAE/100 g) [38]. After two hours, the total phenolic content is stable under different conditions over time. The highest promoted content is observed at 6 bar (A) (48.3 ± 18.6 %) and 9 bar (A) (30.9 ± 13.3 %) at 6 h. However, the ROS content is particularly high at 9 bar (A). Hence this condition can be ruled out. Until now, only limited studies on the phenolic content of *S. cerevisiae* have been carried out. Co-culture of *Lactobacillus* and yeast in extruded brown rice can be used as a functional supplement to provide more bioaccessible antioxidants. Up to 93% increase in total phenolics was reported by Khan et al. [39], while the modality of inoculation is a challenge and needs further investigation. Phenolics can be absorbed from the fruit pomace by yeast [40]. This bio-absorption technique could be used in conjunction with the methods we report here (creating a pressurized fermentation of fruit pomace) to further yeast antioxidant content. 

### 3.4. Reducing Power Measurement

**Figure 6:**
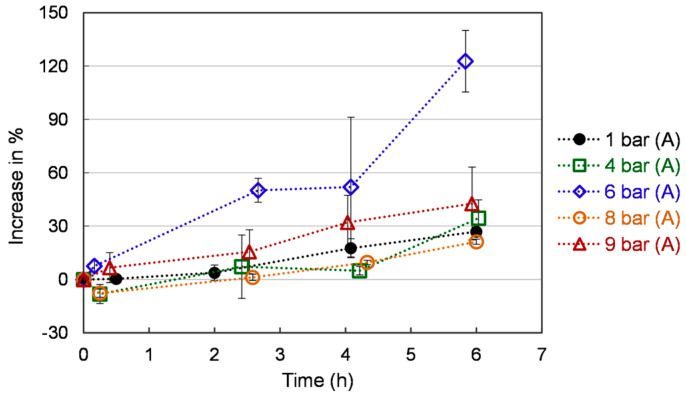
Reducing power of *S. cerevisiae* under different pressures. Each value is expressed as the average ± standard deviation (n = 3).

Figure 6 represents the reducing power as a function of time for the different pressure levels. The reducing power increases gradually throughout the 6 h of the experimental period. A faster rise and a higher level of reducing power were observed in yeast cells under 6 bar (A) compared to that at 8 bar (A) and 9 bar (A). The reducing power of yeast cells ranges globally from 0 to 120 % under 6 bar (A) and from 0 to 30% times under 4, 8, and 9 bar (A). These values are time-dependent but, contrary to ROS, present a monotonic behavior. The reducing properties of yeast and yeast extract are generally associated with the presence of reductones, which have been shown to function as an antioxidant by providing a hydrogen atom to break the free-radical chain. Reductones have also been reported to react with different precursors of peroxides, thus, preventing their formation [41,42]. Thus, we may infer that O_2_ stress-induced reductones expression is possible under specific conditions [43].

### 3.5. Ferrous Ion Chelating Activity

**Figure 7:**
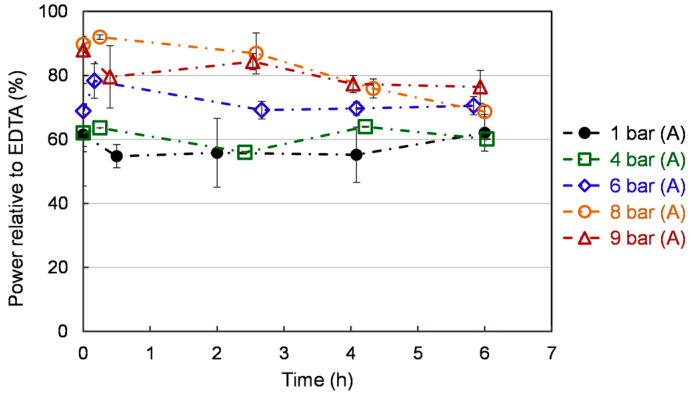
Ferrous ion (Fe2+) chelating activity of *S. cerevisiae* under different pressures. Each value is expressed as the average ± standard deviation (n = 3).

Iron (Fe^2+^) chelators are essential antioxidant compounds as they can retard metal-catalyzed oxidation and also prevent oxidative damage by removing Fe^2+^. Thus, reducing Fe^2+^ may afford protection against oxidative damage by inhibiting the generation of harmful ROS and lipid peroxidation [44]. As shown in Figure 7, ferrous ion (Fe^2+^) chelating activity is stable over time at 1 bar (A) and 4 (A), while a moderate decline is observed at 6, 8, and 9 bar (A). This decrease is lower at 6 bar (A) compared to 8 bar (A). Compared to atmospheric pressure, exposure to higher pressure increases chelating power. The ferrous ion (Fe^2+^) chelating activity in the flat profiles is lower than the others. The 8 bar (A) showed the highest chelating activity at the early cultivation. At 6 bar (A), a mild increase (70%) is exhibited by chelating activity compared to 1 bar (A) and 4 bar (A) (60%). Ferrous ions are commonly extracted in food systems by different solvents. For example, 53.8% Fe^2+^-chelating activity was achieved by methanol of yeast solid-state fermented soybean curd residue [45]. Additional toxic chemicals lead to the cost of a subsequent purification process, which is requested for the application in the field of agri-food or animal feed. 

### 3.6. Antioxidant Activity by β-Carotene-Linoleic Acid Assay

**Figure 8:**
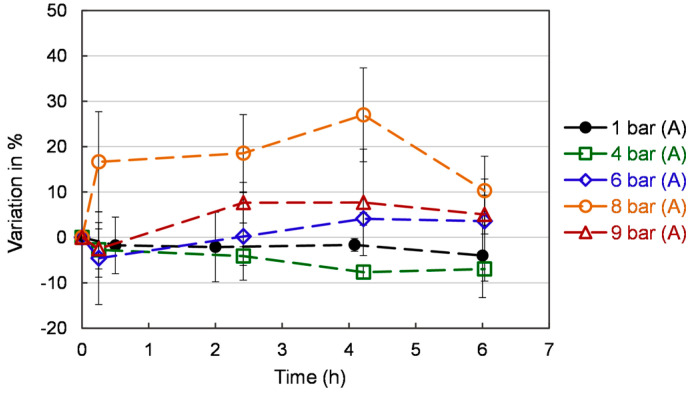
Antioxidant activity by β-carotene-linoleic acid assay of *S. cerevisiae* under different pressures. Each value is expressed as the average ± standard deviation (n = 3).

The effect of different oxygen pressures on antioxidant activity by β-carotene-linoleic acid in yeast cells is shown in Figure 8. Increasing pressure on yeast culture promotes the antioxidant property compared to atmospheric pressure. In the β-carotene bleaching assay, the presence of antioxidants prevents β-carotene bleaching. The oxidation of linoleic acid generates hydroperoxides that can attack β-carotene molecules and cause rapid discoloration of the solution. As one can see, pressure significantly impacts the antioxidant activity, and the value is stable after 2 h. The 6, 8, and 9 bar (A) increased the antioxidant power versus 1 bar (A) and 4 bar (A). However, 9 bar (A) also has an increased ROS content. This is the reason why it should be ruled out. 

## 4. Conclusions

In conclusion, this work reports a novel antioxidant accumulation process in yeast cells. This process is based on increasing oxygen pressure by air resulting in oxidative stress. In order to maintain a stable intracellular redox environment, yeast rose their antioxidant expression through the enzymatic and non-enzymatic pathways. The results obtained clearly shows a positive effect of oxygen pressure on antioxidant power in yeast, which can be summed up as follows:

- In the first stage, the large amount of ROS will lead to a substantial increase in oxidative power, resulting in a net loss of antioxidant power. Thus, it is clear from the above that while the antioxidant power increases, the oxidative power also increases. To maximize the gain in antioxidant power in microorganisms, it is essential to apply hyperbaric conditions to them for a sufficiently long period of time to achieve a decrease in oxidant power while maintaining the gain in antioxidant power.
- To demonstrate the performance of the process, a series of experiments were performed using various operating conditions. Among the investigated conditions, the best results were obtained at 6 bar (A) applied for 2 h. In particular, the manipulation of pressure within a culture bioreactor to generate stress to obtain an increase in the antioxidant power of the microorganisms is easy to implement, easy to plan at the industrial level, and inexpensive.
- In implementing the present process, no additional chemicals, potentially toxic or harmful to the microbial culture, need to be added. Consequently, it is also not necessary to remove this possible chemical product from the medium for the application for which the microorganism is intended, particularly when this application is in the field of agri-food or animal feed. The process, therefore, makes it possible to avoid additional purification costs.

To sum up, this study introduces a novel technique to improve the antioxidant power using stress generation by applying pressure in a microbial culture bioreactor. It can reduce the global operation cost of the accumulation processes and can make it possible to avoid additional purification costs.

## 5. Patents

A patent was published resulting from this work: Pozzobon V., Cui N., Perré P., Procédé pour augmenter le pouvoir antioxidant de microorganisms. FR2109818, CentraleSupélec, 17 September 2021.

## Data Availability

Data are available on demand.
